# Modeling Protein Aggregation and the Heat Shock Response in ALS iPSC-Derived Motor Neurons

**DOI:** 10.3389/fnins.2018.00086

**Published:** 2018-02-20

**Authors:** Emily R. Seminary, Samantha L. Sison, Allison D. Ebert

**Affiliations:** Department of Cell Biology, Neurobiology, and Anatomy, Medical College of Wisconsin, Milwaukee, WI, United States

**Keywords:** C9orf72, SOD1, OPTN, TDP-43, HspB8, HspB1, BAG3, stress granules

## Abstract

Amyotrophic lateral sclerosis (ALS) is a devastating neurodegenerative disorder caused by the selective loss of the upper and lower motor neurons. Only 10% of all cases are caused by a mutation in one of the two dozen different identified genes, while the remaining 90% are likely caused by a combination of as yet unidentified genetic and environmental factors. Mutations in *C9orf72, SOD1*, or *TDP-43* are the most common causes of familial ALS, together responsible for at least 60% of these cases. Remarkably, despite the large degree of heterogeneity, all cases of ALS have protein aggregates in the brain and spinal cord that are immunopositive for SOD1, TDP-43, OPTN, and/or p62. These inclusions are normally prevented and cleared by heat shock proteins (Hsps), suggesting that ALS motor neurons have an impaired ability to induce the heat shock response (HSR). Accordingly, there is evidence of decreased induction of Hsps in ALS mouse models and in human post-mortem samples compared to unaffected controls. However, the role of Hsps in protein accumulation in human motor neurons has not been fully elucidated. Here, we generated motor neuron cultures from human induced pluripotent stem cell (iPSC) lines carrying mutations in *SOD1, TDP-43*, or *C9orf72*. In this study, we provide evidence that despite a lack of overt motor neuron loss, there is an accumulation of insoluble, aggregation-prone proteins in iPSC-derived motor neuron cultures but that content and levels vary with genetic background. Additionally, although iPSC-derived motor neurons are generally capable of inducing the HSR when exposed to a heat stress, protein aggregation itself is not sufficient to induce the HSR or stress granule formation. We therefore conclude that ALS iPSC-derived motor neurons recapitulate key early pathological features of the disease and fail to endogenously upregulate the HSR in response to increased protein burden.

## Introduction

Amyotrophic lateral sclerosis (ALS) is a fatal, adult onset neurodegenerative disorder caused by the loss of the upper and lower motor neurons. Approximately 90% of all cases are sporadic (sALS), likely caused by a combination of as yet unidentified genetic and environmental factors. The remaining cases, familial ALS (fALS), are caused by a mutation in one of the two dozen different genes that have thus far been linked to ALS (Zufiria et al., 2016). Of these, mutations in *C9orf72, SOD1*, or *TDP-43* are the most common, together responsible for at least 60% of fALS (Zufiria et al., 2016). However, the identified genes have varied functions which, together with the inherent heterogeneity of sALS, have made identifying convergent mechanisms of this disease challenging.

Despite this difficulty, a few similarities have been identified between all cases of ALS, including the presence of protein aggregates in the brain or spinal cord (Mizuno et al., 2006). These inclusions typically contain a core set of proteins, including SOD1, TDP-43, and optineurin (OPTN), to name a few. Given the known toxic effects of protein aggregates (Boya et al., 2003; De Kimpe et al., 2013; Yu et al., 2014), attempting to clear these aggregates represents a desirable therapeutic target that could be broadly applicable.

Heat shock proteins (Hsps) are cells' natural defense system against protein aggregation. These chaperone proteins recognize and bind misfolded proteins and either promote their refolding or degradation. Interestingly, it has been shown that Hsps are downregulated during natural aging and various neurodegenerative disorders (Brehme et al., 2014). In accordance with this, a decrease in Hsp70, Hsp40, and HSF1 protein levels in a mouse model of TDP-43-linked ALS and sALS post-mortem tissue has been reported (Chen et al., 2016). Additionally, the protective nature of Hsps in an ALS disease state is further supported by *in vitro* and mouse studies showing an amelioration of disease symptoms by upregulating the heat shock response (HSR) (Kieran et al., 2004; Kalmar et al., 2008; Lin et al., 2013, 2016). However, in contrast to these data, others report increased serum levels of Hsp70 and Hsp90 in ALS patients (Miyazaki et al., 2016). As previous studies have indicated that aberrant and sustained activation of the HSR diminishes its overall function (Roth et al., 2014), it is important to better understand what the expression levels of Hsps are in human ALS motor neurons under physiological conditions and whether these levels are consistent across multiple genetic backgrounds.

Previous reports have shown that motor neurons from ALS mice, postmortem human patients, and iPSCs exhibit protein aggregates (Mizuno et al., 2006; Mackenzie et al., 2007; Kalmar et al., 2008; Maruyama et al., 2010; DeJesus-Hernandez et al., 2011; Lin et al., 2013; Chen et al., 2016; Dafinca et al., 2016; Bhinge et al., 2017), which may contribute to motor neuron loss. As increased levels of insoluble protein triggers the HSR in an effort to degrade and/or refold aberrant proteins (Ananthan et al., 1986; Baler et al., 1992; Morimoto, 1998; Westerheide et al., 2012), we hypothesized that ALS motor neurons have minimal endogenous HSR activation thereby contributing to the presence of protein aggregates. Here, we used motor neurons derived from iPSCs generated from ALS patients expressing mutations in SOD1, C9orf72, and TDP-43. Consistent with previous reports, ALS iPSC-derived motor neurons showed accumulation of insoluble SOD1, TDP-43, and OPTN, but the specific content varied among the different genetic backgrounds. However, the presence of insoluble protein was not sufficient to induce a robust HSR or enhanced stress granule formation in ALS iPSC-derived motor neurons, despite the general ability to induce the HSR with heat stress. Therefore, these data suggest that ALS iPSC-derived motor neurons fail to endogenously upregulate the HSR in response to protein accumulation, which could adversely impact motor neuron health and survival.

## Materials and methods

### iPSC culture

iPSCs were grown on Matrigel (Corning, cat. 354230) coated 6-well plates (VWR, cat. 82050-842) in either mTeSR1 (STEMCell Technologies, cat. 5850) or Essential 8 (Life Technologies, cat. A1517001). Colonies were split every 4–7 days with Versene (Life Technologies, cat. 15040-066) and routinely tested for mycoplasm using the Mycoalert Detection Kit (Lonza, cat. LT07-318). At the time of passage, the remaining wells were harvested to use for filter-trap experiments. For these studies, one SOD1 (N139K) line (Hosoyama et al., 2014), three C9orf72 lines (Sareen et al., 2013), one TDP-43 (M337V) line (Bilican et al., 2012), and three control lines (Ebert et al., 2009; McGivern et al., 2013; Riedel et al., 2014) were used.

### Motor neuron differentiation

Motor neurons were generated by following the protocol described by Maury and colleagues with minor modifications (Maury et al., 2015). Briefly, embryoid bodies (EBs) were generated from monolayer iPSC colonies and transferred to a T25 culture flask. EBs were subjected to various patterning factors for 2 weeks before being dissociated and plated onto Matrigel coated plates for RNA and protein analysis, or Matrigel coated glass coverslips for immunocytochemistry. Motor neurons were allowed to mature for an additional 1–2 weeks before being used for further experiments. For heat shock experiments, plates were wrapped with parafilm and placed in a water bath at 42°C for 1 h. Cells were immediately harvested for RNA and protein analysis.

### Immunocytochemistry

Motor neurons grown on glass coverslips were fixed with 4% Paraformaldehyde for 10 min at room temperature. Cells were permeabilized and blocked with 0.1% Triton and 5% donkey serum for 1 h, and incubated in primary antibody overnight at 4°C or 1 h at room temperature. Secondary antibody was added for 1 h at room temperature. Stained coverslips were blinded and mounted onto glass microscope slides (Fisher, cat. 22-230-891) using Fluoromount-G (Southern Biotech, cat. 0100-01). Antibodies used are listed in Table 1.

**Table 1:** Antibodies.

**Antibody**	**Company**	**Catalog number**
Rabbit α-BAG3	abcam	ab47124
Goat α-ChAT	Millipore	AB144P
Mouse α-G3BP	abcam	ab56574
Rabbit α-HSF1 Phospho S326	abcam	ab76076
Mouse α-HspB1	Cell Signaling	2402
Rabbit α-HspB8	abcam	ab151552
Mouse α-Islet1	DSHB	40.2D6
Rabbit α-Optineurin	abcam	ab151240
Rabbit α-SOD1	abcam	ab13498
Rabbit α-TDP-43	abcam	ab109535
Chicken α-Tuj1	GeneTex	GTX85469
Rabbit α-Tuj1	Covance	MRB-435P

### Microscopy

Images were taken on an upright Nikon E400 microscope and QCapture camera. Light source was a Lumen200 metal arc lamp (Prior Scientific). Pictures of puncta staining were taken with a 100x oil objective, pixel resolution of 0.06 μm/px. All others were taken with a 40x objective, pixel resolution of 0.16 μm/px. Exposure times varied with each target, but were kept consistent when imaging each differentiation. The exposure time ranges per channel are as follows: FITC 700 ms−1 s, TRITC 700 ms−1 s, Cy5 1 s−2 s, DAPI 40–100 ms.

### Image analysis

Quantification was performed in NIS Elements (Nikon). For cell count quantification, cells were counted by a blinded observer. HspB1 and HspB8 staining was quantified by selecting the region of interest, determining the total intensity and then dividing by the total area. Punctate expression was automatically detected in NIS Elements by utilizing the Object Counts module. Intensity and size thresholds were set and kept consistent for each target. Individual neurons were outlined as regions of interests to quantify the number of neurons with puncta as well as the number of puncta per neuron.

### qPCR

Total RNA was isolated with RNeasy Mini Kit (Qiagen, cat. 74104) from cell pellets collected at 4 weeks of total differentiation. 2 μg of RNA was used to generate cDNA according to manufacturer instructions (Promega, cat. A3500). qPCR for HspB1, HspB8, BAG3, and GAPDH was performed using 20 ng cDNA and a Bio-Rad Connect96 Thermocycler with the following cycle conditions: 95°C for 10 min and 40 cycles of 95°C for 10 s and 60°C for 45 s for HspB1, HspB8, and GAPDH. The BAG3 qPCR was performed with the same cycle conditions but with an annealing temperature of 55°C. All signals were normalized to GAPDH and then compared to controls. Primer sequences are listed in Table 2.

**Table 2:** Primer Sequences.

**Target**	**Sequences**	**Citation**
HSPB1 Forward	AAGCTAGCCACGCAGTCCAA	Wang et al., 2013
HSPB1 Reverse	CGGCAGTCTCATCGGATTTT	Wang et al., 2013
HSPB8 Forward	GCCAGAGGAGTTGATGGTGAAGACC	Ospelt et al., 2014
HSPB8 Reverse	CATGTTTGCCAGACACCTCCACG	Ospelt et al., 2014
BAG3 Forward	CATCCAGGAGT GCTGAAAGTG	Li et al., 2017
BAG3 Reverse	TCTGAACCT TCCTGACACCG	Li et al., 2017
GAPDH Forward	GTGGACCTGACCTGCCGTCT	Patitucci and Ebert, 2016
GAPDH Reverse	GGAGGAGTGGGTGTCGCTGT	Patitucci and Ebert, 2016

### Western blot

Protein was isolated by resuspending cell pellets in 1x CHAPS Buffer (Cell Signaling, cat 9852S) followed by three freeze-thaw cycles. The protein concentration of the resulting lysate was determined by a Bradford Assay. 20 μg of protein was loaded into a 4–15% Mini-PROTEAN® TGX Stain-Free™ Protein Gel (Bio-Rad, cat. 4568083), run at 105 V for approximately 70 min, and subsequently transferred to a PVDF membrane (Li-COR, cat. 926-31099) at 105 V for 30 min. The membrane was allowed to dry immediately after transfer for at least 1 h to cross-link the protein before proceeding. REVERT Total Protein Stain (Li-COR, cat. 926-11010) was performed to confirm proper transfer and equal loading. Blots were blocked with a 1:1 dilution of Odyssey Blocking Buffer (Li-COR, cat. 927-50000) and TBS with gentle shaking. Primary antibody was diluted in the same 1:1 dilution plus 0.2% Tween-20 and incubated overnight at 4°C with gentle shaking. Signal was detected by incubating in secondary antibody, diluted in Odyssey Blocking Buffer, TBS, 0.2% Tween-20, and 0.02% SDS, in a light protected box for 30 min at room temperature. Blots were imaged with the Odyssey Scanner (Li-COR). All signals were quantified in Image Studio (Li-COR) and normalized to REVERT Total Protein Stain according to manufacturer instructions. Data are presented as fold change over control. Representative images were converted to grayscale for figures. All antibodies used are listed in Table 1.

### Filter-trap assay

Fourty micrograms of protein was diluted in 1x PBS and 10% SDS, then diluted further in 1x PBS + 0.1% SDS. Protein was loaded onto a Cellulose Acetate Membrane (SterliTech, cat. CA023001) through a dot-blot apparatus and washed with 1x PBS + 0.1% SDS. The membrane was immediately blocked in 5% milk for 1 h and incubated in primary antibody overnight at 4°C with gentle shaking. Signal was detected by WesternBright ECL (Advansta cat. K-12045) after incubating in an HRP-conjugated secondary antibody for 1 h. Densitometry was performed in ImageJ. Representative images were converted to grayscale for figures. Table 1 lists the antibodies used.

### Statistical analysis

A minimum of three independent experiments with a minimum of three biological replicates were performed per experiment. Data were analyzed by performing a one-way ANOVA with Tukey *post-hoc* test or Student's *t*-test, as appropriate. Significance was determined based on a *p* < 0.05.

## Results

### Motor neuron cultures from multiple ALS genotypes do not show decreased viability

**Figure 1:**
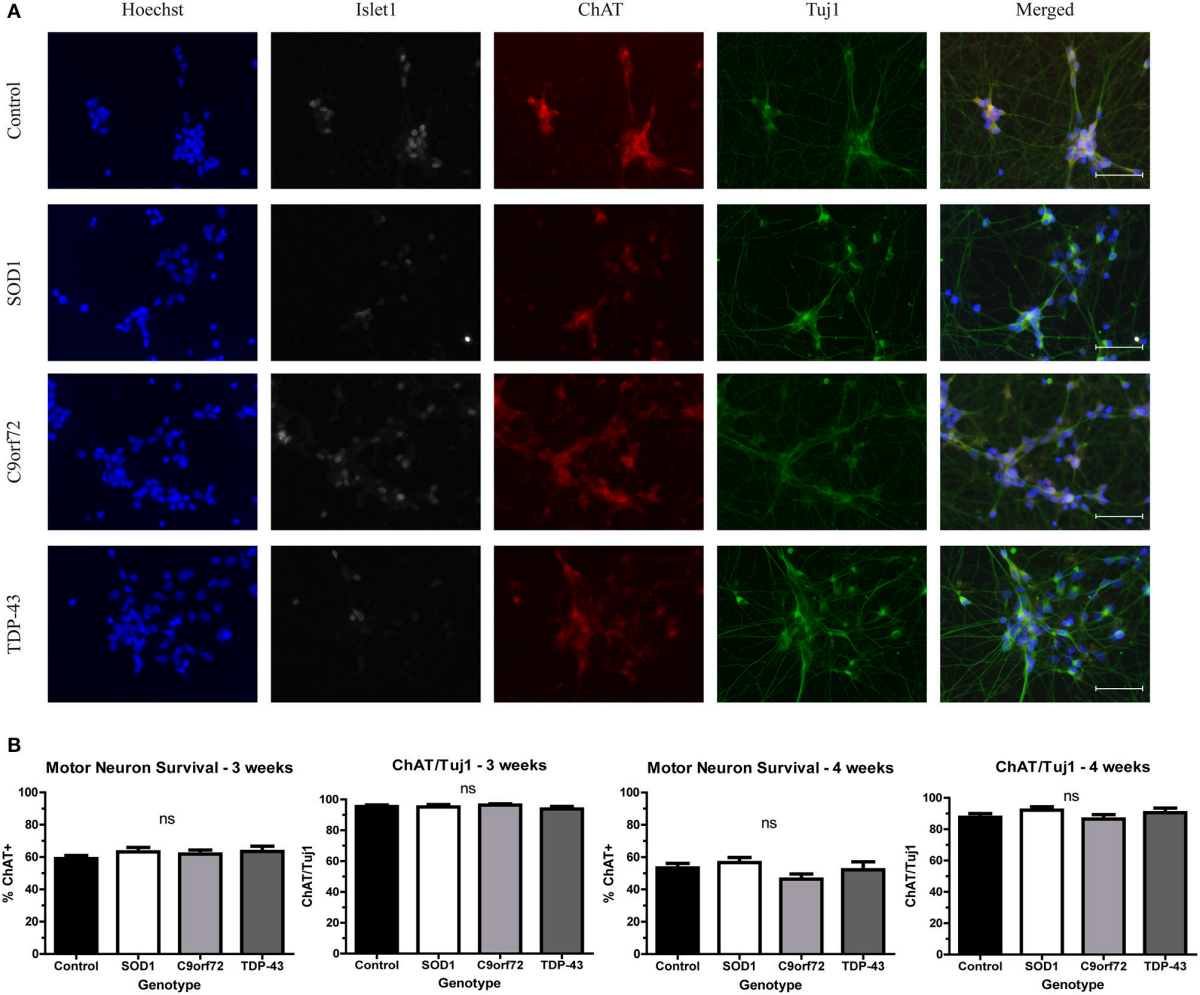
iPSC-derived motor neurons from multiple ALS genotypes do not exhibit reduced viability in culture. **(A)** Representative images at 3 weeks of differentiation of control and ALS motor neurons immunostained for Tuj1 (green), ChAT (red), and Islet1 (white). Nuclei are labeled with Hoechst nuclear dye (blue). Scale bar = 50 μm. **(B)** Quantification shows no difference in the number of motor neurons at 3 or 4 weeks of differentiation. *n* = 3–4. Not significant by one-way ANOVA with Tukey *post-hoc* test.

While motor neuron loss is a key feature of fALS and sALS, previous *in vitro* studies have shown that iPSC-derived motor neurons do not exhibit reduced viability when grown in the absence of astrocytes (Egawa et al., 2012; Almeida et al., 2013; Chen et al., 2014; Devlin et al., 2015). Consistent with previous reports, there was no difference in the number of motor neurons in SOD1, C9orf72, or TDP-43 cultures compared to controls at 4 weeks in culture (Figure 1). Approximately 60% of cells in the cultures were Tuj1+ neurons, with the vast majority of neurons (~95%) expressing the motor neuron markers Islet1 and ChAT (Figure 1) with negligible expression of the astrocyte marker GFAP (data not shown; Maury et al., 2015).

### Divergent solubility of aggregation-prone proteins in ALS motor neurons

**Figure 2:**
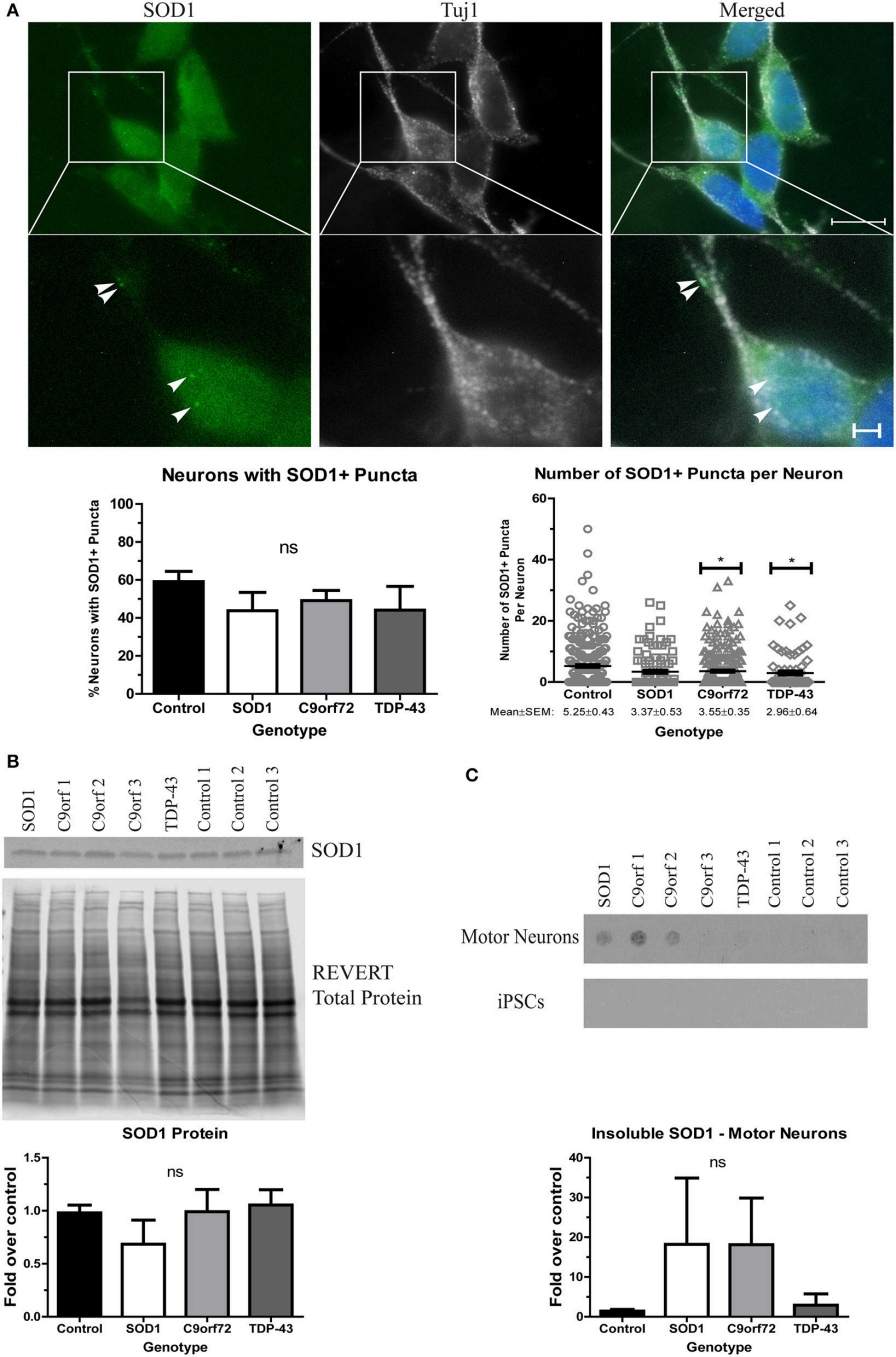
Altered solubility of SOD1 in SOD1 and C9orf72 motor neurons. **(A)** Representative immunocytochemistry images of Hoechst nuclear stain (blue), SOD1 (green), and Tuj1 (white) at 4 weeks of differentiation shows no significant difference in the number of neurons with SOD1+ puncta in ALS motor neurons compared to control (ns, *p* > 0.05 by one-way ANOVA with Tukey *post-hoc* test). C9orf72 and TDP-43 motor neurons exhibited fewer SOD1+ puncta per neuron compared to control (^*^p < 0.05 by one-way ANOVA with Tukey *post-hoc* test). *n* = 3, scale bar = 10 μm (full image) or 2 μm (expanded). **(B)** Western blot for SOD1 shows no significant difference in ALS lines compared to control lines. Data are normalized to REVERT total protein stain and expressed as a fold change over control (ns, *p* > 0.05 by one-way ANOVA with Tukey *post-hoc* test, *n* = 3–6). **(C)** Filter-trap assay shows a trend toward increased levels of insoluble SOD1 in SOD1 and C9orf72 motor neurons but not in iPSC colonies (ns, *p* > 0.05 by one-way ANOVA with Tukey *post-hoc* test). Data are expressed as fold change over controls. *n* = 3.

We next sought to determine whether ALS iPSC-derived motor neuron cultures endogenously develop protein aggregates as this is an important phenotypic hallmark of ALS pathology. While protein aggregation has consistently been observed in mouse models of ALS (Kalmar et al., 2008; Lin et al., 2013; Chen et al., 2016), there has been variability in iPSC models (Devlin et al., 2015; Dafinca et al., 2016; Bhinge et al., 2017). We first asked whether SOD1 aggregates can be detected. Previous studies have reported SOD1+ aggregates in SOD1 iPSC-derived motor neurons (Chen et al., 2014; Bhinge et al., 2017), but TDP-43 and C9orf72 motor neurons have not been evaluated. We found that while there was no difference in the number of neurons with SOD1+ puncta across the different lines, C9orf72 and TDP-43 motor neuron cultures exhibited fewer puncta per neuron compared to control motor neurons (Figure 2A). SOD1 iPSC-derived motor neurons also exhibited a trend toward increased levels of insoluble SOD1 (Figure 2C) and a trend toward decreased levels of soluble SOD1 protein compared to control (Figure 2B). Interestingly, levels of insoluble SOD1 were also trending upwards in C9orf72 motor neurons (Figure 2C), although this phenotype varied among the three independent C9orf72 iPSC lines. These data contrast with the result that C9orf72 motor neurons had fewer SOD1+ puncta per neuron, which suggests that observable puncta may not directly correlate with protein insolubility. Insoluble SOD1 was not detected in any of the iPSC colonies (Figure 2C), which indicates that SOD1 aggregation increases with neuron differentiation.

**Figure 3:**
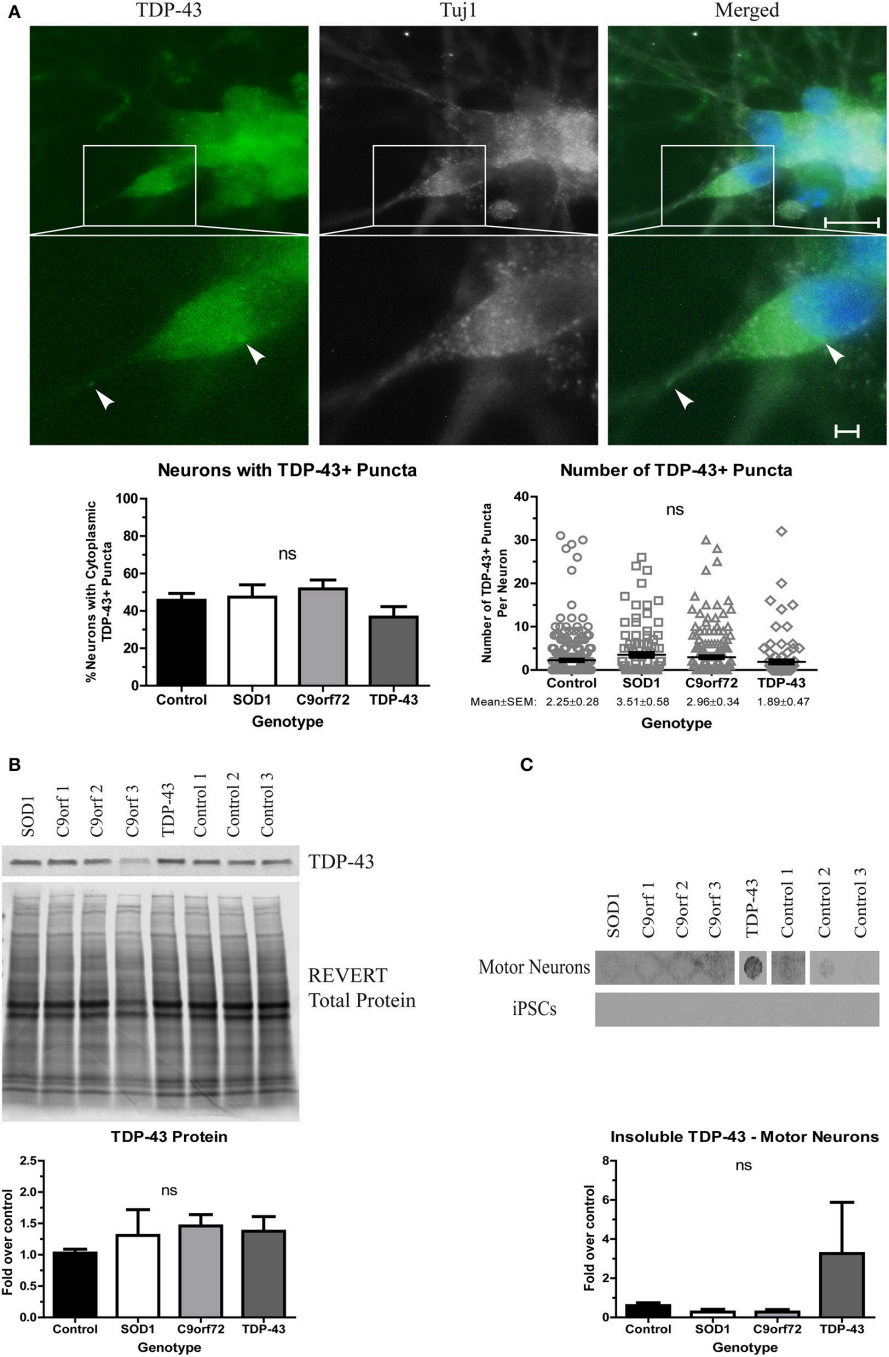
Alterations in TDP-43 solubility in TDP-43 motor neurons. **(A)** Representative images at 4 weeks of differentiation show no change in the number of neurons with cytoplasmic TDP-43+ puncta or the number of TDP-43+ puncta per neuron in ALS motor neuron cultures (ns, *p* > 0.05 by one-way ANOVA). *n* = 3, scale bar = 10 μm (full image) or 2 μm (expanded). **(B)** Western blot for TDP-43 shows no significant difference in protein levels in ALS motor neuron cultures. Data are normalized to REVERT total protein stain and expressed as a fold change over control (ns, *p* > 0.05 by one-way ANOVA, *n* = 3–5). **(C)** Filter-trap assay of TDP-43 shows a trend toward an increase in insoluble levels in TDP-43 motor neurons but not in iPSC colonies (ns, *p* > 0.05 by one-way ANOVA with Tukey *post-hoc* test). Data are expressed as fold change over controls, *n* = 3.

When evaluating TDP-43 aggregation, we found no significant difference in either the number of neurons with cytoplasmic TDP-43+ puncta or the number of puncta per neuron in any of the ALS iPSC lines compared to control (Figure 3A), which is in contrast to a previous report that showed more inclusions in TDP-43 iPSC-derived cultures (Egawa et al., 2012). While all lines showed a trend toward increased soluble levels of TDP-43 compared to control (Figure 3B), these data did not reach significance. However, the TDP-43 motor neurons did exhibit a strong trend toward increased insoluble TDP-43 compared to control (Figure 3C). As with SOD1 protein aggregation, the increase in insoluble protein was most pronounced in the motor neuron cultures (Figure 3C).

**Figure 4:**
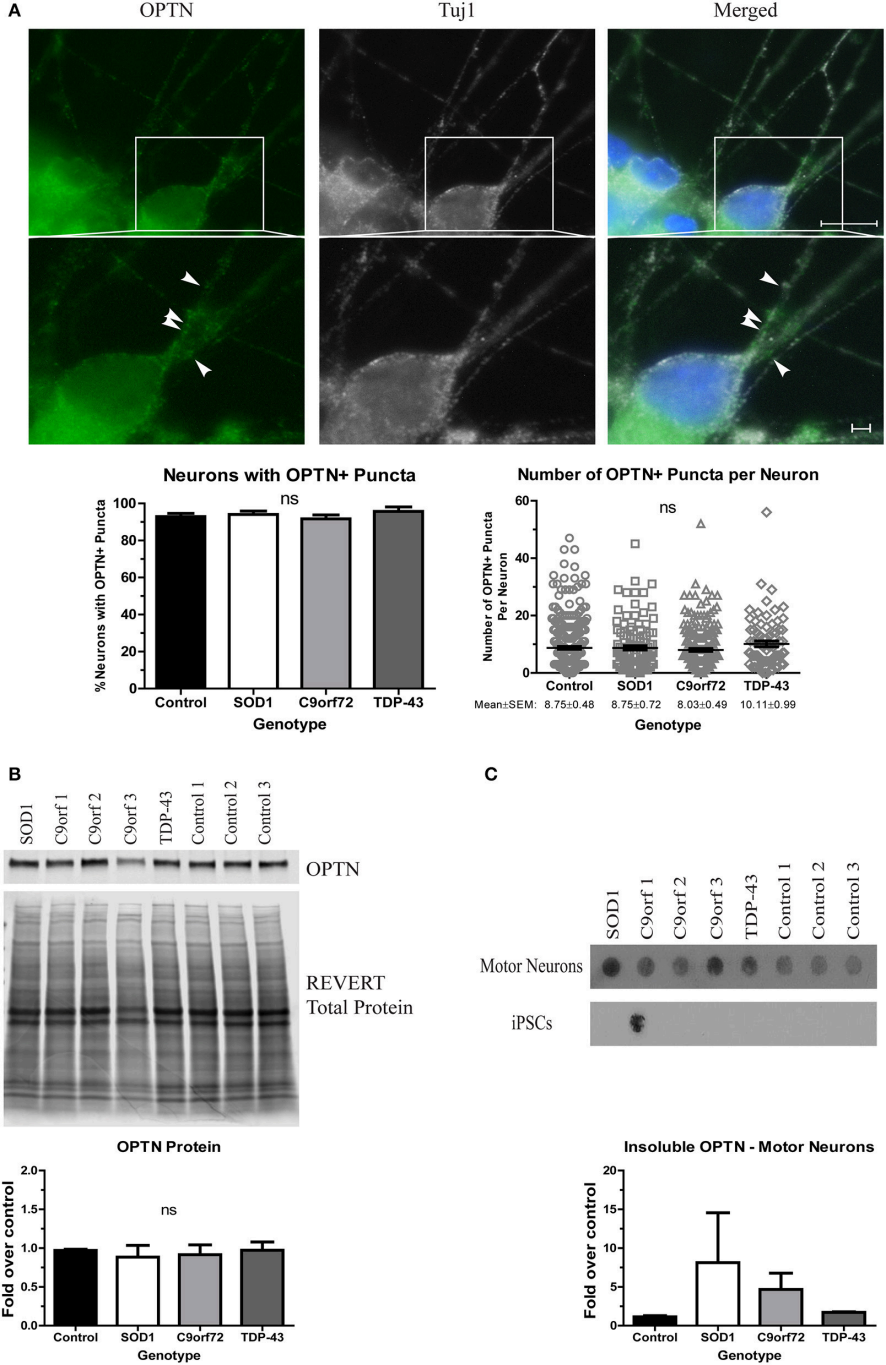
OPTN is insoluble in SOD1 and C9orf72 motor neurons. **(A)** Representative immunocytochemistry images of Hoechst nuclear stain (blue), OPTN (green), and Tuj1 (white) at 4 weeks of differentiation. No difference is seen in the number of neurons with OPTN+ puncta (ns, *p* > 0.05 by one-way ANOVA with Tukey *post-hoc* test). *n* = 3, scale bar = 10 μm (full image) or 2 μm (expanded). **(B)** Total protein levels of OPTN are unchanged by Western blot. Data are normalized to REVERT total protein and expressed as a fold change over control (ns, *p* > 0.05 by one-way ANOVA with Tukey *post-hoc* test). *n* = 3. **(C)** OPTN filter-trap assay shows a trend toward increased insoluble protein in SOD1 and C9orf72 motor neurons (ns, *p* > 0.05 by one-way ANOVA with Tukey *post-hoc* test). Insoluble OPTN was observed in the colonies from one C9orf72 iPSC line, but in no others. Data are expressed as fold change over controls, *n* = 3.

Finally, we asked whether OPTN aggregates in ALS iPSC-derived motor neurons. OPTN is an adaptor protein in autophagy, which, along with the HSR, is a component of the proteostasis network. OPTN can be found in aggregates in both sALS and fALS, and specific mutations have been linked to a small percentage of fALS cases (Maruyama et al., 2010). However, OPTN pathology has not yet been evaluated in iPSC-derived motor neurons. We found no difference in the number of neurons with OPTN+ inclusions in SOD1, TDP-43, or C9orf72 cultures compared to control (Figure 4A). Additionally, the number of OPTN+ puncta per neuron was similar in all lines (Figure 4A). However, whereas soluble protein levels were unchanged across the different lines (Figure 4B), insoluble levels trended higher in SOD1 and C9orf72 motor neuron cultures compared to control cultures, which exhibited a low basal level of insoluble protein (Figure 4C). While one C9orf72 iPSC cell line exhibited increased insoluble OPTN in the undifferentiated state, this phenotype was largely restricted to motor neurons (Figure 4C). Taken together, these data suggest that ALS iPSC-derived motor neurons exhibit accumulation of aggregation-prone proteins, but genetic background does influence protein content and solubility.

### ALS motor neurons fail to fully upregulate the HSR

**Figure 5:**
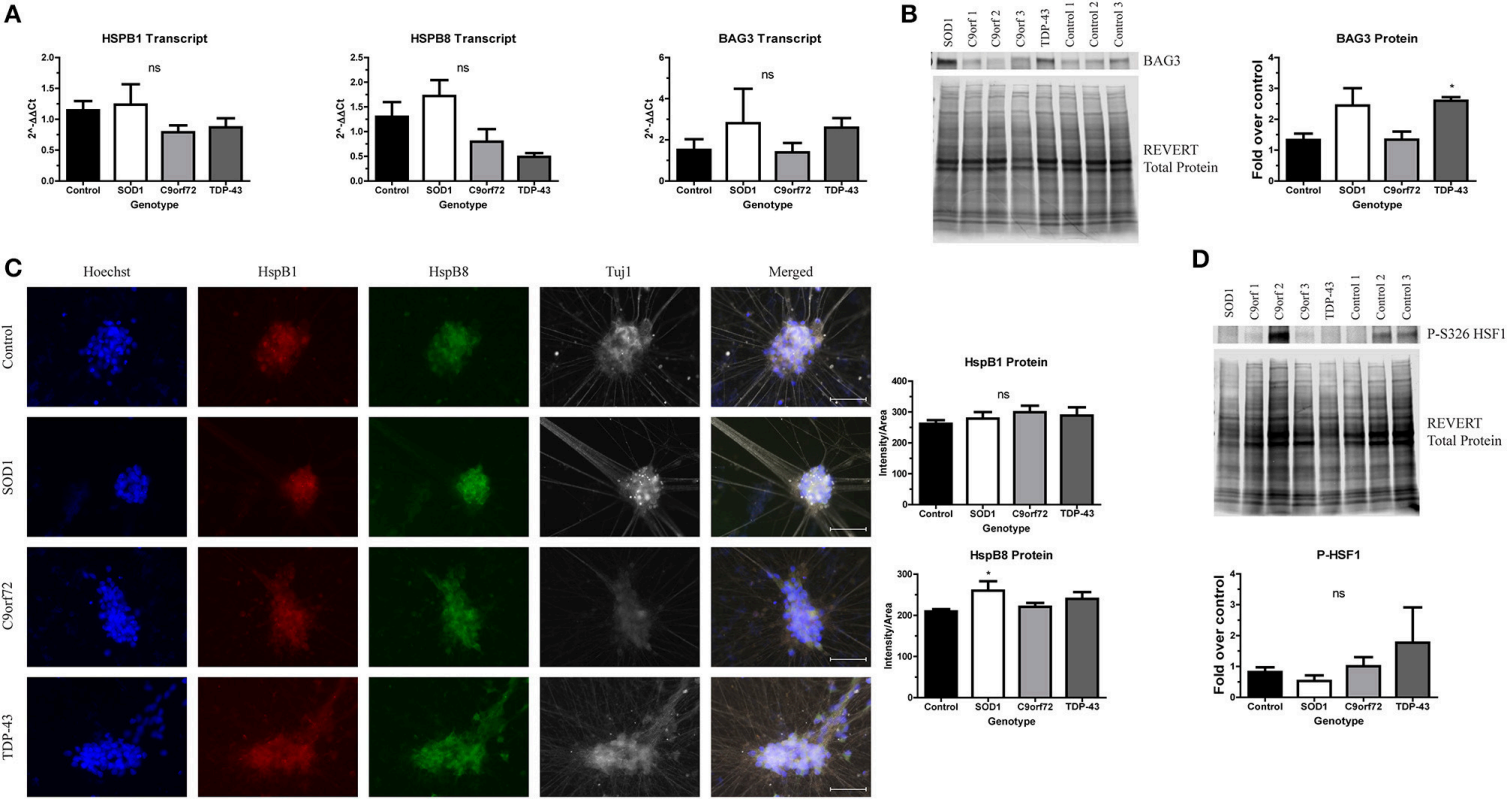
ALS motor neurons have altered expression of HspB1, HspB8, and BAG3. **(A)** BAG3, HspB1, and HspB8 transcript levels are not significantly increased in ALS iPSC-derived motor neurons, *n* = 3–6. **(B)** Western blot for BAG3 shows an increase in TDP-43 motor neurons. Data are normalized to REVERT total protein and expressed as a fold change over control (^*^p < 0.05 by one-way ANOVA with Bonferroni *post-hoc* test, *n* = 3). **(C)** Quantification of HspB1 (green) and HspB8 (red) immunocytochemistry intensity shows a significant increase in protein levels of HspB8 in SOD1 motor neurons (^*^p < 0.05 by one-way ANOVA with Tukey *post-hoc* test, *n* = 3). Scale bar = 50 μm. **(D)** Western blot for phosphorylated HSF1 at S326 shows no significant HSR induction in ALS motor neurons. Data are normalized to REVERT total protein and expressed as a fold change over control (ns, *p* > 0.05 by one-way ANOVA with Tukey *post-hoc* test). *n* = 3.

Protein aggregation is normally prevented by the protein quality control network, which includes the HSR. As such, previous studies have investigated whether inducing the HSR reduces the toxicity of SOD1 or TDP-43 aggregates (Kieran et al., 2004; Lin et al., 2013, 2016; Chen et al., 2016). However, other data indicate that sustained activation of the HSR actually diminishes the function of the protein quality control network (Roth et al., 2014). Since there is an increase in insoluble protein in ALS iPSC-derived motor neurons (Figures 2–4), we next aimed to test whether this could be due to insufficient or aberrant HSR activation. As such, we examined the expression levels of HspB1, HspB8, and BAG3 in ALS iPSC-derived motor neuron cultures (Figure 5). These chaperone proteins were of interest because of previous data linking them to SOD1 (Crippa et al., 2010; Mateju et al., 2017) and TDP-43 pathology (Crippa et al., 2016; Ganassi et al., 2016) in both cell culture and *in vivo* models. Additionally, BAG3 has been shown to complex with HspB1 and HspB8 to promote protein clearance (Ganassi et al., 2016; Rauch et al., 2017). Whereas the transcript levels of HspB1, HspB8, and BAG3 were not significantly altered in C9orf72 motor neurons, we found a trend toward increased BAG3 transcript in SOD1 and TDP-43 motor neurons compared to control motor neurons (Figure 5A). This corresponded to a modest, but significant increase in BAG3 protein levels in TDP-43 motor neurons and a strong trend toward increased protein levels in SOD1 motor neurons compared to control cultures as detected by western blot (Figure 5B). HspB1 and HspB8 protein levels were evaluated by immunocytochemistry intensity quantification, which found no change in HspB1 protein levels in any of the ALS lines compared to control, but a significant increase in HspB8 protein only in SOD1 motor neurons compared to control (Figure 5C). These data indicate that ALS iPSC-derived motor neurons do not have endogenous activation of the HSR in response to increased protein burden.

Considering the modest and incomplete upregulation of the HSR in ALS iPSC-derived motor neurons, we hypothesized that upstream HSR signals may also be blunted. Therefore, we next assessed levels of phosphorylated HSF1 in ALS iPSC-derived motor neurons. HSF1 has been shown to be a master regulator of the HSR (Santoro, 2000; Westerheide et al., 2012). HSF1 is constitutively expressed and sequestered in the cytoplasm by Hsp70, Hsp90, and Hdj1 in its inactive, monomeric form (Morimoto, 1998; Shi et al., 1998). Upon stress and protein aggregation, the chaperones holding HSF1 are recruited to the site of misfolded proteins, releasing HSF1 (Morimoto, 1998; Shi et al., 1998; Santoro, 2000; Westerheide et al., 2012). HSF1 can then trimerize and translocate to the nucleus (Morimoto, 1998; Santoro, 2000). Additionally, HSF1 undergoes a number of post-translational modifications, including phosphorylation at Serine 326, which has been shown to be required for transcriptional activity (Guettouche et al., 2005). As such, expression of the S326 phosphorylated form can be used to determine whether the HSR is robustly activated. However, we detected no significant increase in HSF1 phosphorylation levels in any of the ALS iPSC-derived motor neurons compared to control (Figure 5D), consistent with an incomplete induction of HspB1, HspB8, and BAG3. Therefore, these data indicate that the observed increase in insoluble protein burden is not sufficient to activate the HSR in ALS iPSC-derived motor neurons.

**Figure 6:**
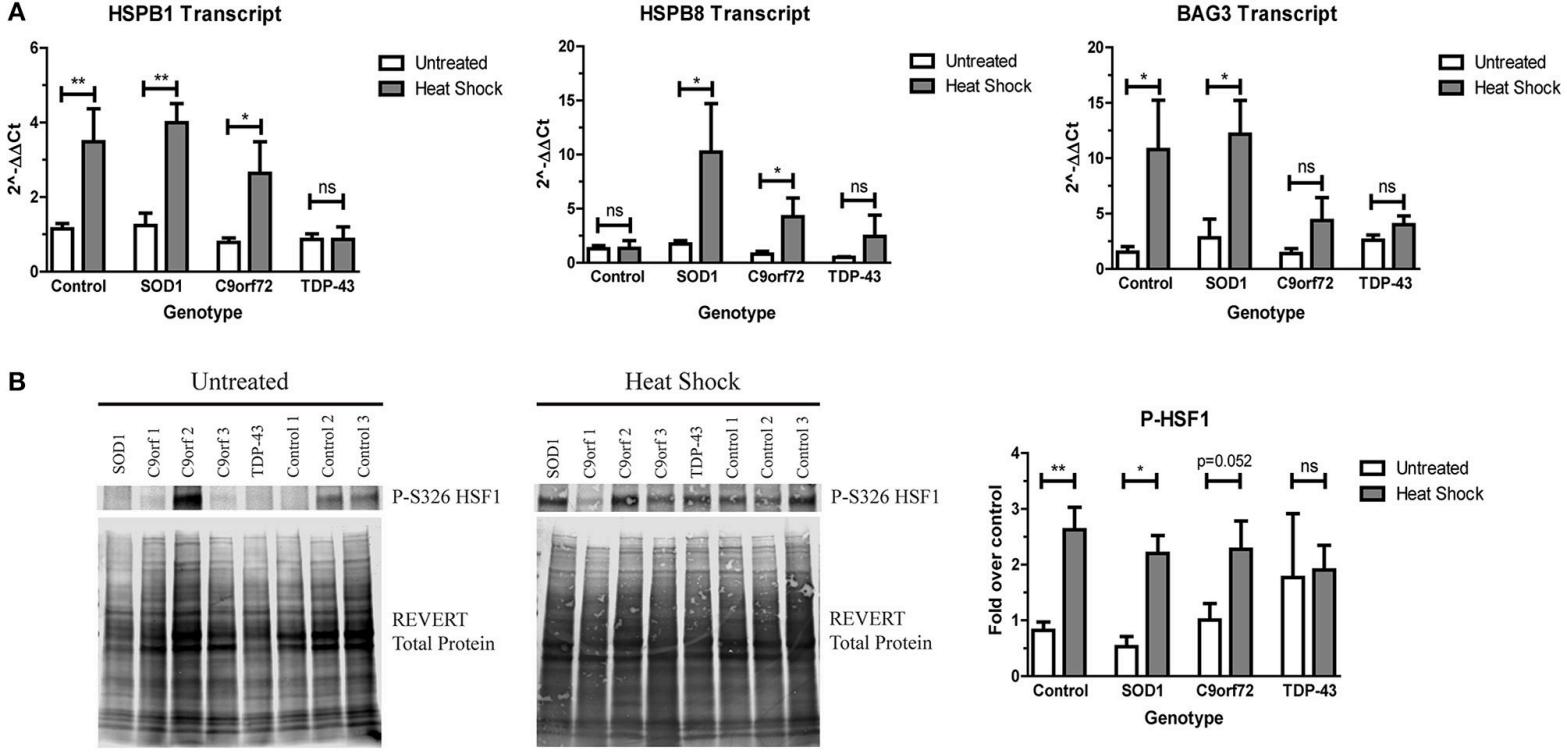
SOD1 and C9orf72 motor neurons induce the HSR after acute heat stress. **(A)** SOD1 and C9orf72 motor neurons have increased transcript levels of HspB1, HspB8, and BAG3 compared to their untreated samples after 1 h of heat shock. Data are normalized to untreated controls (^**^p < 0.01, ^*^p < 0.05 by Student's *t*-test). *n* = 3–4. **(B)** Western blot for phosphorylated HSF1 at S326 shows a significant induction in the HSR in control and SOD1 motor neuron cultures compared to their untreated samples after 1 h of heat shock. Untreated western blot from Figure 5D is shown again for ease of comparison. Data are normalized to REVERT total protein stain and expressed as a fold change over untreated control samples. (^**^p < 0.01, ^*^p < 0.05 by Student's *t*-test). *n* = 3–4.

Since ALS iPSC-derived motor neurons do not robustly activate the HSR in response to increased protein aggregation, we next sought to determine if ALS motor neurons were capable of upregulating the HSR at all. Therefore, we incubated ALS and control iPSC-derived motor neurons at 42°C for 1 h to heat stress the cells. With the exception of HspB8, control motor neurons showed robust induction of HspB1 and BAG3 transcript (Figure 6A) and phosphorylated HSF1 protein (Figure 6B) following heat stress. Similarly, heat stressed SOD1 motor neurons significantly induced transcript levels of HspB1, HspB8, and BAG3 (Figure 6A), as well as exhibited increased phosphorylated HSF1 protein levels compared to the unstressed state (Figure 6B). C9orf72 motor neurons also showed increased HspB1, HspB8, and BAG3 transcript levels following heat stress, although to a lesser extent than the control or SOD1 motor neurons (Figure 6A). Similarly, C9orf72 motor neurons exhibited increased levels of phosphorylated HSF1, although this increase was just below significance (Figure 6B). Interestingly, the TDP-43 motor neurons did not significantly induce any of the measured HSR components after heat shock (Figure 6), suggesting an intrinsic deficiency in the ability of these motor neurons to induce this protein quality control pathway. Together these data suggest that ALS iPSC-derived motor neurons may be able to activate the HSR under certain conditions, but that insoluble protein burden is not a sufficient signal to do so and genetic background may influence overall response.

### ALS motor neurons do not exhibit enhanced stress granule formation

**Figure 7:**
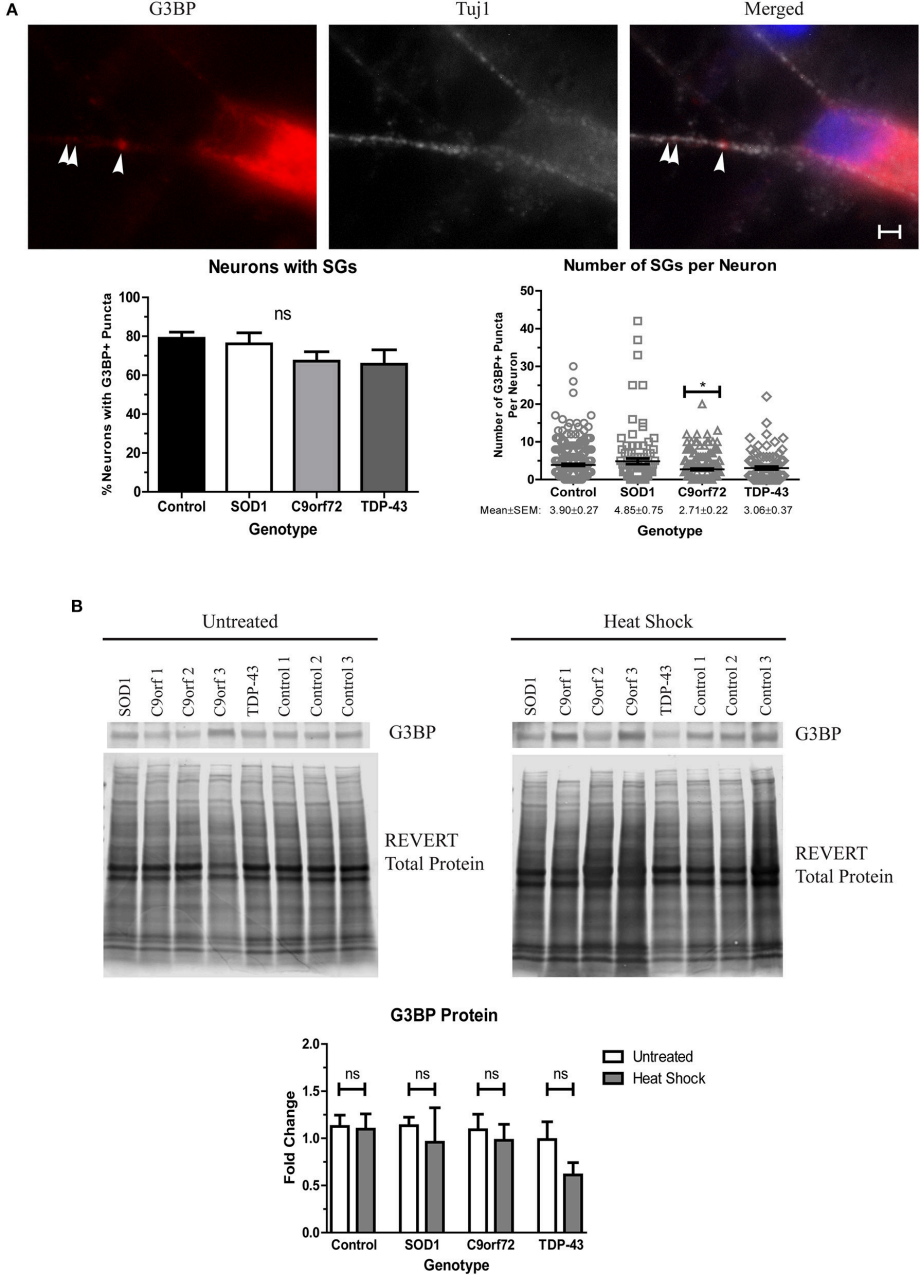
ALS motor neurons do not show enhanced stress granule formation. **(A)** Immunocytochemistry of Tuj1 (white) and G3BP (red) shows no difference in the number of neurons with at least one G3BP+ puncta, but C9orf72 motor neurons exhibit fewer G3BP+ puncta per neuron (^*^p < 0.05 by one-way ANOVA with Tukey *post-hoc* test). *n* = 3, scale bar = 2 μm. **(B)** Western blot for G3BP shows no difference in protein levels in untreated or heat shocked motor neurons (ns, *p* > 0.05 by Student's *t*-test). Data are normalized to REVERT total protein stain and expressed as a fold change over untreated control samples.

Stress granules (SGs) traditionally sequester mRNAs and RNA-binding proteins, like TDP-43, during acute cellular stress into membrane-less compartments (Kedersha et al., 1999). After stress alleviation, SGs disassemble and translation resumes (Kedersha et al., 1999). However, under prolonged stress, misfolded proteins, including SOD1, can accumulate into SGs which can alter SG dynamics, leading to insolubility (Ganassi et al., 2016; Mateju et al., 2017). Additionally, several SG components, including eIF3 and TIA-1, have been found in aggregates in post-mortem ALS tissue (Liu-Yesucevitz et al., 2010), and a recent report has identified TIA-1 as a rare causative mutation for fALS (Mackenzie et al., 2017). We therefore sought to determine whether SGs spontaneously form in ALS iPSC-derived motor neurons as an indication of whether ALS motor neurons upregulate stress pathways as a response to increased insoluble protein. We found that while the staining pattern for the SG marker G3BP was primarily diffuse, the majority of neurons had at least one G3BP+ puncta, with the average number of G3BP+ puncta ranging ~3–5 per neuron (Figure 7A). Interestingly, C9orf72 motor neurons exhibited a modest but significant decrease in the number of SGs per neuron (Figure 7A), which may indicate a deficiency in the ability of C9orf72 motor neurons to induce this protective mechanism. There was no overall change in the protein levels of G3BP in the ALS lines compared to control (Figure 7B), and following heat stress, we also did not detect a change in G3BP protein levels in any sample compared to the unstressed condition (Figure 7B). These data are in contrast to previous studies (Mateju et al., 2017) and suggest that acute heat stress may not be sufficient to induce stress granule formation in the iPSC system. Taken together, these data suggest that ALS iPSC-derived motor neurons lack the capabilities to fully activate stress response systems despite increased insoluble protein burden.

## Discussion

ALS is a highly complex neurodegenerative disease that has neither a cure nor an effective therapy. Despite first being described in the late 1800s, the cause of sALS is still unknown. Additionally, two dozen different genes with varying prevalence and protein functions have been identified as causative for fALS. Given this heterogeneity, it is imperative that any convergent mechanisms and phenotypes be identified in order to develop therapies that would be applicable to the greatest number of patients. Previous studies using *in vitro* and *in vivo* model systems have focused mainly on SOD1, TDP-43, and in recent years C9orf72-linked ALS, as mutations in these three genes represent the most prevalent causes of fALS. However, these studies have limitations. The SOD1 and some TDP-43 mouse models were generated by over-expressing the mutant form of the human proteins, which may not accurately represent the human condition. Multiple C9orf72 mouse models have been made to date, but results have been mixed as each mouse exhibits a different phenotype. A C9orf72 knock-out mouse showed no motor deficits, but did exhibit an immune phenotype and an enlarged spleen (O'Rourke et al., 2016). Alternatively, two BAC C9orf72 models did not develop ALS despite the presence of RNA foci and dipeptide repeat proteins that are characteristic of this mutation (O'Rourke et al., 2015; Peters et al., 2015), while a third BAC mouse did exhibit motor deficits and reduced survival, consistent with an ALS disease state (Liu et al., 2016). Numerous other studies use non-motor neuron cell types that rely on mutant protein over-expression in potentially non-disease-relevant cell types. In contrast, iPSCs offer a unique human model system to study endogenous cellular processes associated with ALS in disease-relevant cell types (Dimos et al., 2008). Additionally, iPSCs are more amenable to concurrently studying multiple genetic backgrounds, which will be crucial for identifying convergent disease mechanisms.

Even though motor neuron loss is a key pathological feature of ALS, iPSC-derived motor neuron cultures do not exhibit a cell death phenotype when grown in the absence of astrocytes (Figure 1), (Dimos et al., 2008; Egawa et al., 2012; Almeida et al., 2013; Chen et al., 2014; Devlin et al., 2015). However, ALS iPSC-derived motor neurons do recapitulate other features of ALS. Specifically, we detected increased levels of insoluble SOD1, TDP-43, and OPTN protein levels in differentiated motor neurons (Figures 2–4) but, each genotype had different components and characteristics. For instance, the SOD1 and C9orf72 motor neurons, but not TDP-43 motor neurons, had increased insoluble SOD1 compared to control motor neurons, (Figure 2C). SOD1 protein aggregation was not observed in any of the iPSC lines in the undifferentiated state suggesting that protein burden increases with terminal differentiation. Although previous studies have shown that SOD1 motor neurons have an abundance of insoluble SOD1 protein (Chen et al., 2014; Bhinge et al., 2017), TDP-43 and C9orf72 iPSC-derived motor neurons had not previously been evaluated for SOD1 expression levels. On average the C9orf72 lines exhibited a trend toward increased insoluble levels of SOD1 (Figure 2C), yet this phenotype varied across the multiple lines. Since each line has approximately the same number of expanded repeats (Sareen et al., 2013), it is possible that there are other disease modifiers contributing to this phenotype that varies in the population. Additionally, it is important to note that only one SOD1 line and one TDP-43 line was used for these studies. As such, utilizing multiple iPSC-lines to take into account population variance will be necessary for future studies.

We also detected TDP-43 pathology in TDP-43 motor neurons. As evidenced by the increase in insoluble protein levels (Figure 3C), TDP-43 has seemingly aggregated in the TDP-43 motor neurons, which is consistent with a previous report (Egawa et al., 2012). Interestingly, while post-mortem tissue from C9orf72 patients show TDP-43+ aggregates (DeJesus-Hernandez et al., 2011), we do not observe an increase in insoluble TDP-43 levels in C9orf72 iPSC-derived motor neuron cultures (Figure 3C). This is perhaps because TDP-43 pathology in C9orf72 ALS is likely a secondary phenotype initiated by the dipeptide repeats that are characteristic of C9orf72-linked pathology (Khosravi et al., 2017). Although the iPSC-derived motor neurons were carried out to four weeks in culture, TDP-43 protein aggregation and pathology might become pronounced with extended times in culture.

OPTN can often be found in protein aggregates in post-mortem ALS samples and is one of the many rare mutations linked to fALS (Maruyama et al., 2010). Importantly, OPTN is an autophagic adaptor protein, and therefore a part of the proteostasis network. Thus, any alterations in OPTN levels, solubility, or localization could have deleterious effects on the neuron's ability to clear protein aggregates. However, OPTN pathology has not previously been evaluated in ALS iPSC-derived motor neuron cultures. Although there was no evidence of OPTN aggregation in TDP-43 motor neurons, increased insoluble OPTN protein levels were observed in SOD1 and C9orf72 motor neurons compared to controls (Figure 4C). Importantly, with the exception of one undifferentiated C9orf72 iPSC-line that showed insoluble OPTN, insoluble protein was most pronounced in differentiated motor neurons. Together, these data are supported by a recent report showing that SOD1, TDP-43, and OPTN are supersaturated in motor neurons, and therefore are especially susceptible to aggregation (Ciryam et al., 2017). As such, although genetic background may alter the specific content of aggregation-prone proteins, motor neurons may not be able to efficiently handle the protein load resulting in motor neuron malfunction and loss.

The proteostasis network, which includes the HSR, ubiquitin proteasome system, and autophagy, functions to prevent protein aggregation. Beyond motor neuron loss, protein aggregation is one of the only pathological similarities that has been identified across ALS patients. Therefore, it is possible that upregulating one or more components of the proteostasis network may be a potential therapy for many cases of ALS. As such, previous studies have determined that inducing the HSR by either overexpressing HSR components or utilizing pharmacological agonists reduces the number and toxicity of protein aggregates in both SOD1 and TDP-43 ALS cell culture and mouse models (Kieran et al., 2004; Kalmar et al., 2008; Crippa et al., 2010, 2016; Lin et al., 2013, 2016; Chen et al., 2016; Ganassi et al., 2016). However, previous work has also shown that aberrant activation of the HSR can diminish the overall efficacy of protein refolding and breakdown (Roth et al., 2014); interestingly human brain samples from patients with Parkinson's disease, Huntington's disease, and Alzheimer's disease all show aberrant HSR activation (Brehme et al., 2014) indicating that this could be an important feature of neurodegeneration. We found that SOD1, TDP-43, and C9orf72 motor neurons have minimal and/or incomplete HSR activation in response to physiological protein burden (Figure 5). HspB1 and HspB8 are both a part of the small chaperone protein family that recognizes misfolded proteins. Additionally, mutations in both genes have been linked to distal hereditary motor neuropathy (Echaniz-Laguna et al., 2017), further indicating their role in motor diseases. BAG3, a co-chaperone protein, can complex with both HspB1 and HspB8 to aid in protein clearance (Ganassi et al., 2016; Rauch et al., 2017). We found that although TDP-43 motor neurons have increased expression of BAG3 compared to controls (Figures 5A,C), there was not a concurrent increase in HspB1 or HspB8 (Figures 5A,B). Similarly, we found that SOD1 motor neurons showed an induction in HspB8 expression compared to control (Figures 5A,B), but only a trend toward higher BAG3 and no increase in HspB1 (Figures 5A,B). Interestingly, the C9orf72 iPSC-derived motor neurons did not show any significant increase in BAG3, HspB8, or HspB1 compared to control (Figure 5). These data, along with no significant change in phosphorylated HSF1 levels in any ALS line compared to controls (Figure 5D), suggest that increased insoluble protein burden is not sufficient to fully activate the HSR in ALS motor neurons.

Perhaps most interestingly, ALS motor neurons exhibited divergent induction of the HSR in response to heat shock. When cultured at 42°C for 1 h, SOD1 motor neuron cultures were able to robustly induce the HSR compared to the non-stressed condition (Figure 6). Given the apparent benefit of this pathway in clearing SOD1 aggregates (Kieran et al., 2004; Kalmar et al., 2008; Crippa et al., 2010; Lin et al., 2013), these data support the use of HSR agonists in the treatment of SOD1-linked ALS, such as the HSR inducing agent arimoclomol (Kieran et al., 2004; Kalmar et al., 2008, 2014). Although to a lesser degree than control and SOD1 motor neurons, C9orf72 iPSC-derived motor neurons were able to induce this pathway after heat shock (Figure 6). These results also suggest that HSR agonists may be beneficial for C9orf72-associated ALS, although a particularly effective agonist may be required given the more modest induction. TDP-43 motor neurons, however, did not induce the HSR after heat shock as evidenced by the lack of significant increase in HspB1, HspB8, or BAG3 transcript levels nor increased HSF1 phosphorylation (Figure 6). It is possible that TDP-43 motor neurons have an intrinsic deficit in their ability to activate the HSR and HSR agonists may not be an effective therapy for TDP-associated ALS, but more research is needed since only one TDP-43 iPSC line was used. Curiously, we did not observe an induction of HspB8 transcript levels in control motor neurons after heat shock (Figure 6A). Given that these qPCR experiments were performed on the same samples as those used for HspB1 and BAG3, it is unlikely that there was a problem with the heat shock itself. However, as HspB8 is temperature sensitive, it is unclear why it was not induced in any of the three control lines.

SGs have primarily been linked to FUS-linked ALS as FUS is an RNA-binding protein that can be found in normal granules. However, various SG components have previously been found in post-mortem protein aggregates that are immunopositive for TDP-43 (Liu-Yesucevitz et al., 2010), and HspB1 and HspB8 are recruited to aberrant SGs (Ganassi et al., 2016; Mateju et al., 2017). While dynamic SGs are a part of the normal stress response, prolonged exposure to misfolded proteins can cause SGs to lose this dynamism which in turn leads to more insoluble aggregates (Mateju et al., 2017). Interestingly, we saw no change in the number of neurons with at least one SG in ALS motor neuron cultures (Figure 7A). This is in contrast to previous reports that have shown increased numbers of SGs in *in vitro* models of SOD1-, TDP-43-, or C9orf72-linked ALS (Liu-Yesucevitz et al., 2010; Dafinca et al., 2016; Mateju et al., 2017). However, the majority of these previous studies demonstrating an increase in SG formation were observed under exogenous stress conditions, including heat shock. Yet, we did not see a significant increase in protein levels of G3BP after heat shock (Figure 7B). It is therefore possible that acute heat shock is not a sufficient stress to induce SG formation in ALS iPSC-derived motor neurons, and that aging or DNA damage may be necessary. This is corroborated by studies that have shown an induction in the number of SGs in FUS iPSC motor neuron cultures exhibiting significant DNA damage (Higelin et al., 2016).

iPSCs represent a valuable human motor neuron model system in order to further study the role of the HSR in ALS pathogenesis, as different genetic backgrounds and variable protein burden may influence the overall cellular response. These data show that despite maintaining viability, physiological levels of mutant proteins in SOD1, TDP-43, and C9orf72 iPSC-derived motor neurons induced insoluble protein accumulation, but that the protein burden varied across different genotypes. Moreover, insoluble protein may accumulate in ALS motor neurons over time due to a minimal endogenous activation of the HSR rather than aberrant overexpression. As such, these data lend further support to the use of HSR agonists as a potential therapeutic strategy for ALS. However, as there were also differences in expression of HSR components across the different ALS iPSC lines, more research is needed to parse out how the individual mutated proteins could impact the overall function of the protein quality control system in ALS.

## Author contributions

ES and AE designed the experiments. ES performed experiments, analyzed data, and wrote the manuscript. SS assisted with data analysis and iPSC maintenance. AE assisted with data analysis and wrote the manuscript. All authors reviewed and approved of the final document.

### Conflict of interest statement

The authors declare that the research was conducted in the absence of any commercial or financial relationships that could be construed as a potential conflict of interest.
